# Yersinia pseudotuberculosis Bacteremia Complicated by Rhabdomyolysis

**DOI:** 10.7759/cureus.23192

**Published:** 2022-03-15

**Authors:** Masayoshi Kusunoki, Ryuichi Ohta, Nozomi Nishikura, Chiaki Sano

**Affiliations:** 1 Department of Emergency Medicine, Shimane Prefectural Central Hospital, Izumo, JPN; 2 Communiy Care, Unnan City Hospital, Unnan, JPN; 3 Community Care, Unnan City Hospital, Unnan, JPN; 4 Community Medicine Management, Shimane University Faculty of Medicine, Izumo, JPN

**Keywords:** rural hospital, rhabdomyolysis, primary care, foodborne disease, yersiniosis, yersinia pseudotuberculosis

## Abstract

*Yersinia pseudotuberculosis *is a rare pathogen that causes yersiniosis, a foodborne disease that has become more prevalent in recent years*. *Yersiniosis commonly causes gastrointestinal symptoms; however, bacteremia can be the primary clinical finding. Here, we report the case of an 83-year-old man who presented with fever and fatigue and was diagnosed with *Y. pseudotuberculosis* bacteremia. Gastrointestinal findings were absent at the time of admission. His condition was complicated by rhabdomyolysis, which was self-limiting and resolved spontaneously. This case reveals that fever may be the only clinical sign of invasive yersiniosis and that it can be complicated by rhabdomyolysis. Clinicians should consider *Y. pseudotuberculosis* as a potential causative pathogen in patients with a fever of unknown origin and rhabdomyolysis.

## Introduction

*Yersinia *species are gram-negative nonsporulating coccobacilli belonging to the Enterobacteriaceae family. Out of 17 species in the *Yersinia* genus, three are pathogenic for humans, namely, *Yersinia pestis* that causes the plague, *Yersinia enterocolitica*, and *Yersinia pseudotuberculosis* [1]. *Y. enterocolitica* is the primary cause of yersiniosis associated with gastroenteritis and mesenteric adenitis, which often mimics appendicitis [1-3]. *Y. pseudotuberculosis* is known as a rather rare causative pathogen of yersiniosis. However, yersiniosis caused by *Y. pseudotuberculosis* has become more prevalent worldwide in recent years and has caused several outbreaks in Japan and Europe over the past few decades [2,3]. According to a study conducted in the United States, the mean annual incidence of *Y. enterocolitica* and *Y. pseudotuberculosis* infection between 1996 and 2007 was 3.5 and 0.04 per million persons, respectively [4]. Compared with *Y. enterocolitica* infection, patients with *Y. pseudotuberculosis* infection have a higher median age (6 versus 47 years), hospitalization rate (30% versus 72%), and mortality rate (1% versus 11%) [4]. Although yersiniosis can infect anyone, children under the age of five years, persons with reduced immunity, and older adults have an increased risk of infection [1]. The main sources of yersiniosis in humans include raw or undercooked pork, unpasteurized milk, and drinking water [1,5]. Both *Y. enterocolitica* and *Y. pseudotuberculosis* can proliferate at cold temperatures as low as 0°C, and *Y. pseudotuberculosis* can grow at refrigeration temperatures (0-4°C). The median incubation period of *Y. pseudotuberculosis* is eight days (range: 4-25 days) [3,6].

Here, we present the case of an 83-year-old man who developed *Y. pseudotuberculosis* bacteremia complicated by rhabdomyolysis one week after eating raw pork dumplings. The absence of gastrointestinal findings on admission made the diagnosis process difficult and caused the delay in diagnosis. Yersiniosis caused by *Y. pseudotuberculosis* is rare but its incidence may be underestimated; moreover, the knowledge of its epidemiology is limited. To our knowledge, this is the first clinical case report describing *Y. pseudotuberculosis* bacteremia with the complication of rhabdomyolysis. This case illustrates that gastrointestinal findings can be absent on presentation during invasive *Y. pseudotuberculosis* infection. The learning points from this case are the following: detailed history-taking including dietary history is important even when abdominal symptoms are absent, and *Y. pseudotuberculosis* bacteremia can be a possible differential diagnosis when a febrile patient is accompanied by rhabdomyolysis.

## Case presentation

An 83-year-old man presented to the internal medicine ambulatory setting with fatigue and fever that had started on the day of the visit. He was living home alone because his wife had been hospitalized for a stroke a month previously. This had put him in a situation of having to manage all the domestic chores including food preparation during the past month. His medical histories included atrial fibrillation, mitral valve regurgitation, hypertension, and hyperlipidemia, which were being treated with rivaroxaban 15 mg per day, bisoprolol 2.5 mg per day, irbesartan 50 mg per day, and ezetimibe 10 mg per day, respectively. He had a history of an appendectomy at a young age. On presentation, his temperature was 39.7°C with a pulse rate of 95 beats/minute and blood pressure of 130/82 mmHg. He was alert and oriented. His oxygen saturation was 99% when breathing room air and his respiratory rate was 24 breaths/minute. On clinical examination, he had Levine III/VI holosystolic heart murmur, which was best heard at the apex. His breath sounds were normal. He did not report any gastrointestinal symptoms including abdominal discomfort, nausea, diarrhea, or hematochezia. On physical examination, his abdomen was soft and there was no localized tenderness. His face was flushed, but he had no skin rash elsewhere on his body. He had not consumed any alcohol before the presentation. He had no localized signs of muscle injury. His lower legs were warm and mildly edematous.

The laboratory tests on admission showed a normal white blood cell count of 7,900/µL, C-reactive protein of 8.54 mg/dL, and a creatinine kinase (CK) level of 1,509 U/L, which increased to a peak of 7,511 U/L on day three post-admission (Table 1).

**Table 1:** The patient’s laboratory results

Parameter (units)	Day 1	Day 3	Day 9	Reference range
White cell count (×10^3^/µL)	7.90	3.30	3.30	3.8–10.4
Hemoglobin (g/dL)	11.8	12.8	10.7	13.6–16.9
Platelet count (×10^3^/µL)	96	89	157	152–324
Aspartate aminotransferase (U/L)	87	342	98	5–37
Alanine aminotransferase (U/L)	39	105	77	6–43
Blood urea nitrogen (mg/dL)	29.3	27.5	19.5	9–21
Creatinine (mg/dL)	0.97	0.90	0.83	0.6–1.0
Creatine kinase (U/L)	1,509	7,511	374	57–240
C-reactive protein (mg/dL)	8.54			< 0.30

Aspartate aminotransferase (AST)-dominated transaminitis was observed on day three, which can most likely be explained as the result of rhabdomyolysis.

**Figure 1:**
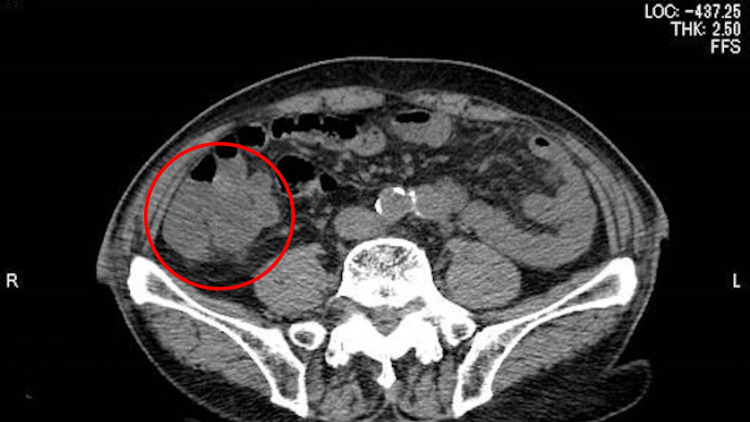
The abdominal computed tomography showing the edematous lesions in the small intestine and colon (red circle).

Electrocardiography did not show any ischemic changes, and there was no evidence of an electrolyte imbalance, such as hypokalemia or hyperkalemia. Hematology showed mild thrombocytopenia (96,000/µL) and mild anemia (hemoglobin: 11.8 g/dL). Urinalysis showed occult hematuria (red blood cells: >50/HPF) and proteinuria, but no bacteria were detected. An abdominal ultrasound showed no abnormal findings in the kidney, biliary tract, and abdominal cavity, except for a mildly distended inferior vena cava (25 mm). Chest radiography showed moderate cardiomegaly but no lung consolidation or pleural effusion. There were no abnormal findings in transthoracic echocardiography except for previously diagnosed mitral regurgitation. Computed tomography (CT) revealed a small amount of fluid collection and mucosal edema, mainly in the ileocecal area, but no abscess formation in the peritoneal space, and no mesenteric lymphadenopathy (Figure 1).

**Figure 2:**
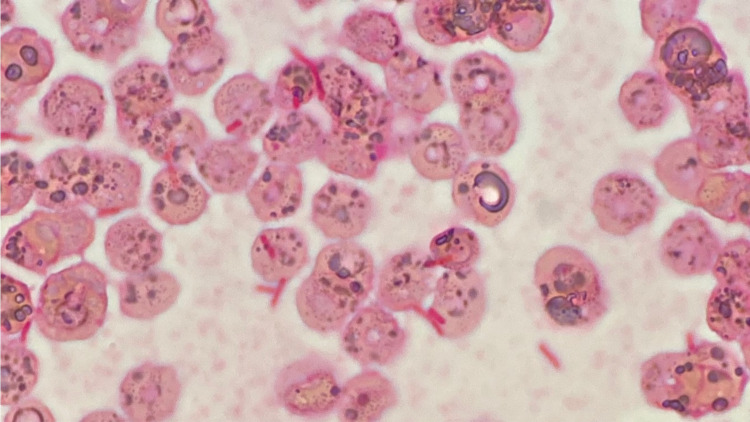
The gram stain of the patient’s blood culture.

The initial working diagnoses included bacterial enteritis, infective endocarditis, and bloodstream infection of unknown origin. Based on the biochemistry and imaging findings, the urinary tract, respiratory tract, and hepatobiliary tract were considered less likely to be the sources of infection. Infective endocarditis was difficult to rule out at the time of admission, but the patient did not satisfy any of modified Duke’s criteria [7]. Although CT revealed mucosal edema and fluid collection in the colon, we were hesitant to diagnose bacterial enteritis because of the absence of gastrointestinal physical findings. The patient was tentatively diagnosed with a bloodstream infection of unknown primary origin complicated by toxin-induced rhabdomyolysis. After the sputum, urine, and blood culture samples had been taken, the empiric ceftriaxone was started against gram-negative rods (GNRs). We did not conduct a stool culture at this stage because there were no gastrointestinal findings such as abdominal pain, diarrhea, or hematochezia on presentation. On day two post-admission, a detailed history-taking from the family members of the patient revealed that three days before admission, the patient had eaten raw pork dumplings without knowing that they were supposed to be cooked. This additional information prompted us to consider the possibility of foodborne disease. Thus, we obtained stool culture from the patient, which showed “no growth,” possibly due to the prior administration of antibiotics. The blood culture taken before administration of antibiotics turned positive for GNRs after 48 hours (Figure 2), which were later identified as *Y. pseudotuberculosis*.

From the history-taking and the laboratory results, including blood culture, we finally diagnosed the patient’s condition as *Y. pseudotuberculosis* bacteremia complicated by rhabdomyolysis. Despite the elevated CK level (as high as 7,511 U/L), he did not complain of any myalgia or abdominal pain during the admission period. We considered the possibility of drug-induced rhabdomyolysis as a differential diagnosis, thus we discontinued ezetimibe and irbesartan on admission. Despite the discontinuation of these medications, CK continued increasing until day three. To rule out the organic diseases in the intestinal tract, we conducted a colonoscopy, which did not show any abnormal findings, except for the appendectomy scar. The causative pathogen was assumed to have entered the bloodstream from the intestinal tract due to the consumption of raw pork dumplings. As the blood culture result showed that *Y. pseudotuberculosis* was sensitive to both ceftriaxone and ampicillin, antibiotics were de-escalated from ceftriaxone to ampicillin on day five post-admission and continued until day 14.

The patient recovered well after the administration of antibiotics and was discharged on day 15. The rhabdomyolysis was self-limiting and asymptomatic, requiring no additional treatment. Although it caused temporary glomerulonephritis resulting in hematuria and proteinuria, his renal function was maintained during the admission period. He was followed up one month after discharge and did not develop any secondary complications such as reactive arthritis.

## Discussion

This case shows that *Y. pseudotuberculosis* can cause bacteremia complicated by rhabdomyolysis. In this patient, the absence of abdominal symptoms on admission made the diagnosis challenging. An important lesson from this case is the importance of detailed clinical history-taking about a patient’s food intake, even in the absence of abdominal symptoms.

*Y. pseudotuberculosis* infection may not cause abdominal pain because of its specific pathophysiology. It can manifest as a severe systemic inflammatory syndrome known as Far East scarlet-like fever (FESLF). In Japan and Russia, several outbreaks of FESLF caused by *Y. pseudotuberculosis* infection have been reported in the past few decades [8]. Studies suggest that there is a geographical heterogeneity in the virulence factors between European and Far Eastern *Y. pseudotuberculosis* strains [8]. *Y. pseudotuberculosis* has also been implicated in the pathogenesis of Kawasaki disease due to its similar clinical presentation [9,10]. While infection with the European strain occurs in the form of self-limiting gastroenteritis, infection with the Far Eastern strain does not necessarily cause abdominal symptoms [8]. In this case, the absence of abdominal findings can be explained by the pathophysiology of FESLF. Clinicians should be aware that infection of *Y. pseudotuberculosis*, especially the Far Eastern strain that occurs in Japan and Russia, can present with systemic inflammatory symptoms rather than gastroenteritis.

Rhabdomyolysis is a rare complication of yersiniosis which can trigger various serious complications. One of the most important complications of rhabdomyolysis is acute renal failure, which has been reported to occur in 16.5% of cases [11]. In the presented case, rhabdomyolysis was self-limiting and recovered without any specific treatment because severe complications such as acute renal failure did not occur. Yersiniosis caused by *Y. pseudotuberculosis* can present with various systemic symptoms, including fever, abdominal pain, back pain, joint pain, and erythema, while diarrhea is less frequent [3]. Its secondary complications include intestinal perforation, subacute obstruction syndrome, intussusception, and acute renal failure [12,13].

Additionally, *Y. pseudotuberculosis* bacteremia can cause septic arthritis [14,15]. However, a review of the literature did not reveal any previous reports of rhabdomyolysis as a complication of *Y. pseudotuberculosis* infection. The causes of rhabdomyolysis are multifold, including drugs, trauma, and infection. Singh and Scheld [16] reported that *Legionella*, *Streptococcus*, and *Salmonella *species are the most common bacterial pathogens associated with rhabdomyolysis. A recent study regarding community-acquired bacterial sepsis revealed that gram-negative pathogens were more frequently associated with rhabdomyolysis than gram-positive pathogens, but *Y. pseudotuberculosis* was not reported as a causative pathogen [17]. Several pathogenic mechanisms of rhabdomyolysis due to sepsis have been proposed in previous studies, including direct muscle invasion, toxin generation, and muscle hypoxia [17,18]. Studies have shown that *Yersinia *species produce toxins that play a role in the colonization of the intestine [19]. Therefore, it is possible that the rhabdomyolysis in this patient was caused by toxin generation.

Drug-induced rhabdomyolysis is another possible differential diagnosis in our case. It is possible that some of his medications contributed as a provocative factor of rhabdomyolysis. However, the continuation of CK elevation even after the discontinuation of the possible causative medications (ezetimibe and irbesartan) at the point of admission makes this differential diagnosis less likely. The Naranjo adverse drug reaction probability scale is useful to classify the probability that an adverse event is related to specific drug therapy or not [20]. In our case, the total Naranjo score was 1 (definite if the overall score is 9 or greater, probable for a score of 5-8, possible for 1-4, and doubtful if the score is 0), which is classified as possible. Overall, it is insufficient to conclude the presented case of rhabdomyolysis is caused by his medications alone due to the low Naranjo score of 1. Although direct muscle invasion is another possible cause, there was no evidence of organic lesions such as colon cancer or abscess formation on CT.

## Conclusions

*Y. pseudotuberculosis* is a rare but important cause of gastroenteritis. To our knowledge, this is the first reported case of *Y. pseudotuberculosis* infection complicated by rhabdomyolysis. Clinicians should consider *Y. pseudotuberculosis* as a possible causative pathogen in the differential diagnosis of patients with fever of unknown origin and rhabdomyolysis. As gastrointestinal signs and symptoms may be absent, detailed history-taking regarding food intake can be the key to the diagnosis of yersiniosis caused by *Y. pseudotuberculosis*.
